# In Memoriam: David E.R. Sutherland

**DOI:** 10.3389/ti.2025.14674

**Published:** 2025-05-06

**Authors:** Rainer W. G. Gruessner

**Affiliations:** Department of Surgery, State University of New York, New York, NY, United States

**Keywords:** pancreas transplantation, islet transplantation, In memoriam, obituary for Dr. David Sutherland, David Sutherland

Dr. David Sutherland, considered the “father” of pancreas and islet transplantation, died peacefully in the early morning hours of 25 March 2025. As one of the most preeminent pioneers in the field of transplantation he epitomized the best of humanity, humility, empathy, integrity, and competence in all of medicine.

**FIGURE 1:**
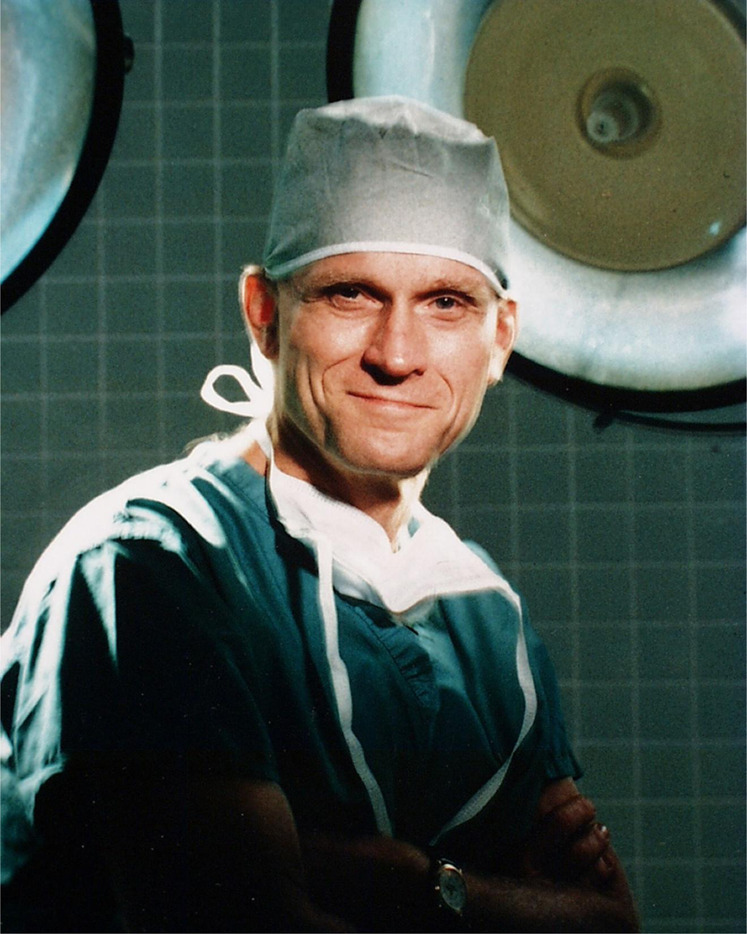
David E. R. Sutherland, MD, PhD. Reproduced with permission from “David E.R Sutherland, MD, PhD” by Rainer W.G. Gruessner and Angelika C. Gruessner, licensed under CC BY 4.0.

David Elmer Richard Sutherland (“DERS”, Figure 1) was born on 25 December 1940, in St. Paul, MN. Upon completion of medical school, surgery residency and transplantation fellowship, all at the University Minnesota, and medical service in the Vietnam war, he stayed on the faculty of the Department of Surgery from 1976 until his retirement in 2009. He served his lifelong institution as the Chief of the Division of Transplantation and the Director of the Schulze Diabetes Institute. In his honor, an endowed chair was established at the University of Minnesota.

His scientific career began in the laboratories of Robert Alan Good (1922–2003), who performed the first successful non-twin human bone marrow transplant and Richard Carlton Lillehei (1927–1981), who performed the world’s first successful pancreas transplant. Sutherland’s early research work focused on the immunological role of the thymus, Peyer’s patches, and appendix resulting in his first publications as a 23-year-old student in the journals *Nature* and *Lancet*. More than 1,000 peer-reviewed articles would follow over the years primarily focusing on all aspects of beta-cell replacement therapies.

Sutherland never considered pancreas and islet transplantation as competing fields, but rather as complementary treatment options in an all-inclusive, comprehensive beta-cell replacement strategy. This explains his treatment shifts from solid-organ to cellular transplantation and *vice versa*, based on the best approach for an individual patient.

His early focus on both pancreas and islet transplantation from living donors, initially with his mentor and chairman, Dr. John S. Najarian, was much more successful than from deceased donors. From the scientific perspective, and before the advent of advanced laboratory tests, Sutherland’s most important immunological finding was that type 1 Diabetes Mellitus is an autoimmune disease that did recur in the twin donor pancreas graft when no immunosuppression was given; and did not recur when standard immunosuppression was administered.

During his 35-year tenure at the University of Minnesota, David Sutherland directed the world’s oldest and largest pancreas and islet transplant programs. He was instrumental to a myriad of surgical “firsts” including the now-called “Sutherland” technique of spleen preservation in patients undergoing distal pancreatectomy, islet auto-transplantation after total pancreatectomy for chronic pancreatitis, and the first successful split-pancreas transplant.

For his many seminal contributions to beta-cell replacement through transplantation, Sutherland received many honors and awards during his distinguished career. He served as the President of the American Society of Transplant Surgeons (1990); of the Cell Transplantation Society (1994); of the International Pancreas and Islet Transplant Association (1995); and of The Transplantation Society (2002). He received honorary doctorates and honorary memberships from institutions around the world and, in 2012, the (Sir Peter) Medawar Prize, the world’s highest dedicated award for the most outstanding contribution in the field of transplantation [1].

However, his distinguished career and unparalleled contributions do justice to only part of David Sutherland’s personality. Equally important, he was an inspiring human being who cared deeply for his patients, suffered tremendously with them in case of setbacks, and relished their successes. He performed transplants in thousands of diabetic patients, many of whom became insulin-independent and dialysis-free free for the rest of their lives. Altruistic by nature, he was a living kidney donor himself. He trained scores of transplant surgeons and physicians from all over the world who admired his humane qualities, pioneering vision, tireless passion and wonderful work ethics. He became a beloved teacher, mentor and surgeon who laid the foundation for the field of pancreas and islet transplantation as we know it. His own curiosity, ingenuity, fearlessness and willingness to think outside the box are legendary; as he once said, “true scholars don’t practice evidence-based medicine, they perform evidence-gathering medicine.” He set a high bar for excellence just by leading by example.

Because he was so helpful and instrumental to numerous careers, many of his trainees moved on to become directors of large pancreas transplant programs, chiefs of transplantation, or department chairs. He earned tremendous respect and admiration from his peers, patients and students alike–while remaining a truly humble, modest, compassionate, principled and easily approachable human being with a great sense of humor. His gracious personality by giving everyone a fair chance simply bred the highest esteem and loyalty. Despite becoming one of the greatest surgeons of the second half of the 20th century, he never forgot his Minnesota roots, nor his many interests in nonmedical fields such as American history and literature, classical music, baseball and horticulture.

We send our grateful sentiments and our condolences and prayers to his wife Vanesa and his family. The transplant community at large is indebted to David Sutherland.
